# Intended outcome expands in time

**DOI:** 10.1038/s41598-017-05803-1

**Published:** 2017-07-24

**Authors:** Mukesh Makwana, Narayanan Srinivasan

**Affiliations:** Centre of Behavioural and Cognitive Sciences, University of Allahabad, Allahabad, 211002 India

## Abstract

Intentional agents desire specific outcomes and perform actions to obtain those outcomes. However, whether getting such desired (intended) outcomes change our subjective experience of the duration of that outcome is unknown. Using a temporal bisection task, we investigated the changes in temporal perception of the outcome as a function of whether it was intended or not. Before each trial, participants intended to see one of two possible outcomes but received the intended outcome only in half of the trials. Results showed that intended outcomes were perceived as longer than unintended outcomes. Interestingly, this temporal expansion was present only when the intended outcome appeared after short action-outcome delays (250 ms-Exp 1 and 500 ms-Exp 2), but not when it appeared after long action-outcome delay (1000 ms-Exp 3). The effect was absent when participants did not intend and performed instruction-based action (Exp 4). Finally, Exp 5 (verbal estimation task) revealed that intention induced temporal expansion occurs via altering the gating or switch mechanism and not the pacemaker speed. Results are explained based on intention-induced pre-activation resulting in extended temporal experience. Our study not only suggests inclusion of intention as a potential factor influencing time perception but also indicates a close link between intentional binding and the intention induced temporal expansion of its outcome.

## Introduction

Human beings are intentional agents. Intention not only guides our action to achieve the desired goal but also alters our perception^1, 2^. What one sees is not solely determined by the bottom up sensory inputs but in fact is modulated by what one desires or intends to see. For example, a bottle-of-water was perceived as closer, when participants intended to drink it compared to when they did not^3^. Similarly, a tool-in-hand was perceived closer only when participants intended to use it rather than just holding it^4^. Given that perceptual space and time are strongly linked^5–7^ and intention influences spatial perception of the intended event, there is a need to study the effect of *“intention”* (i.e. “what one wants”) on time perception.

In the present study, we sought to investigate whether the duration of action outcome is perceived differently as a function of it being intended or unintended. If yes, then what could be its underlying mechanism? While no study has directly investigated the effect of intention on the perceived duration of the outcome, many studies have investigated the effect of intentional or voluntary action on the time between an action and its outcome^8–11^. An example is the *Intentional binding (IB)* effect^8–11^ in which the perceived time of voluntary action and the perceived time of its outcome are shifted towards each other, such that the interval between the two events is compressed.

**Figure 1:**
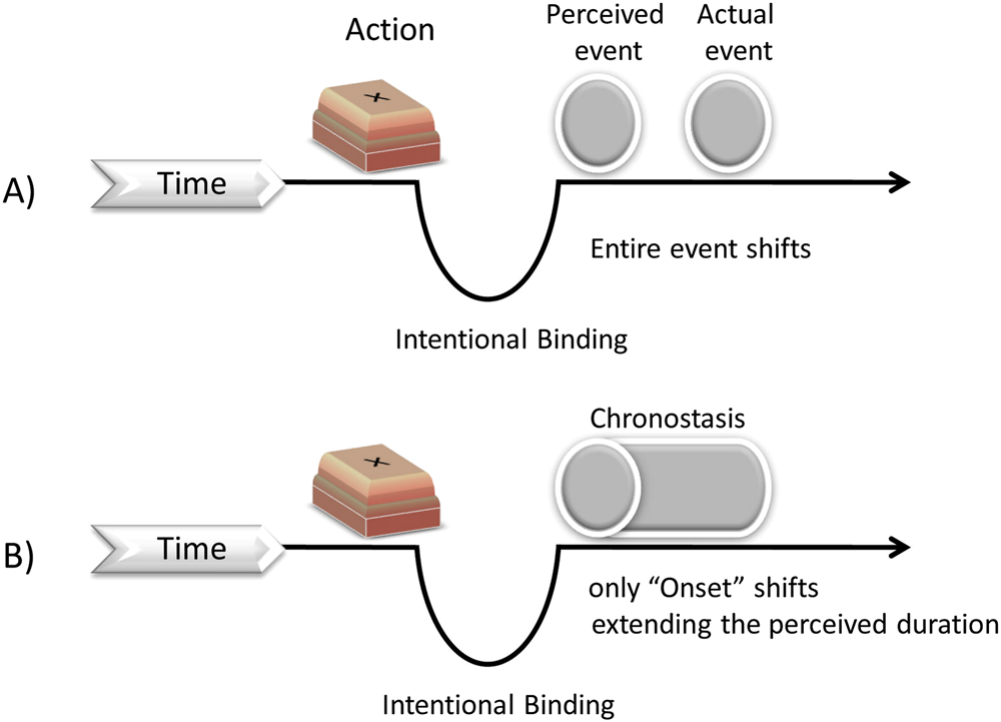
Pictorial representation depicting (**A**) *Intentional binding* where the “entire event shifts” towards the action, and (**B**) where only the “onset shifts” towards the action leading to temporal expansion of the outcome. Temporal expansion associated with actions is also referred as *chronostasis* ^18, 19^.

Many models and explanations have been proposed to explain IB^12–15^. Among these is the sensory-motor recalibration model^15^, which suggests that the brain recalibrates the interval between the action and its outcome such that it shifts the outcome time towards the action. Although this proposal could explain both IB and reversal of temporal order (which they found in their study), it is still unclear during such shifting whether the entire outcome or only the onset shifts towards the action. We believe that studying the duration perception of the outcome would also provide insight in answering this question. If the entire outcome shifts towards the action, then one should not get any temporal expansion of the desired outcome. However, if only the onset shifts towards the action then one should get the temporal expansion of the outcome (see Fig. 1) potentially linking IB with duration perception of the desired outcome.

**Figure 2:**
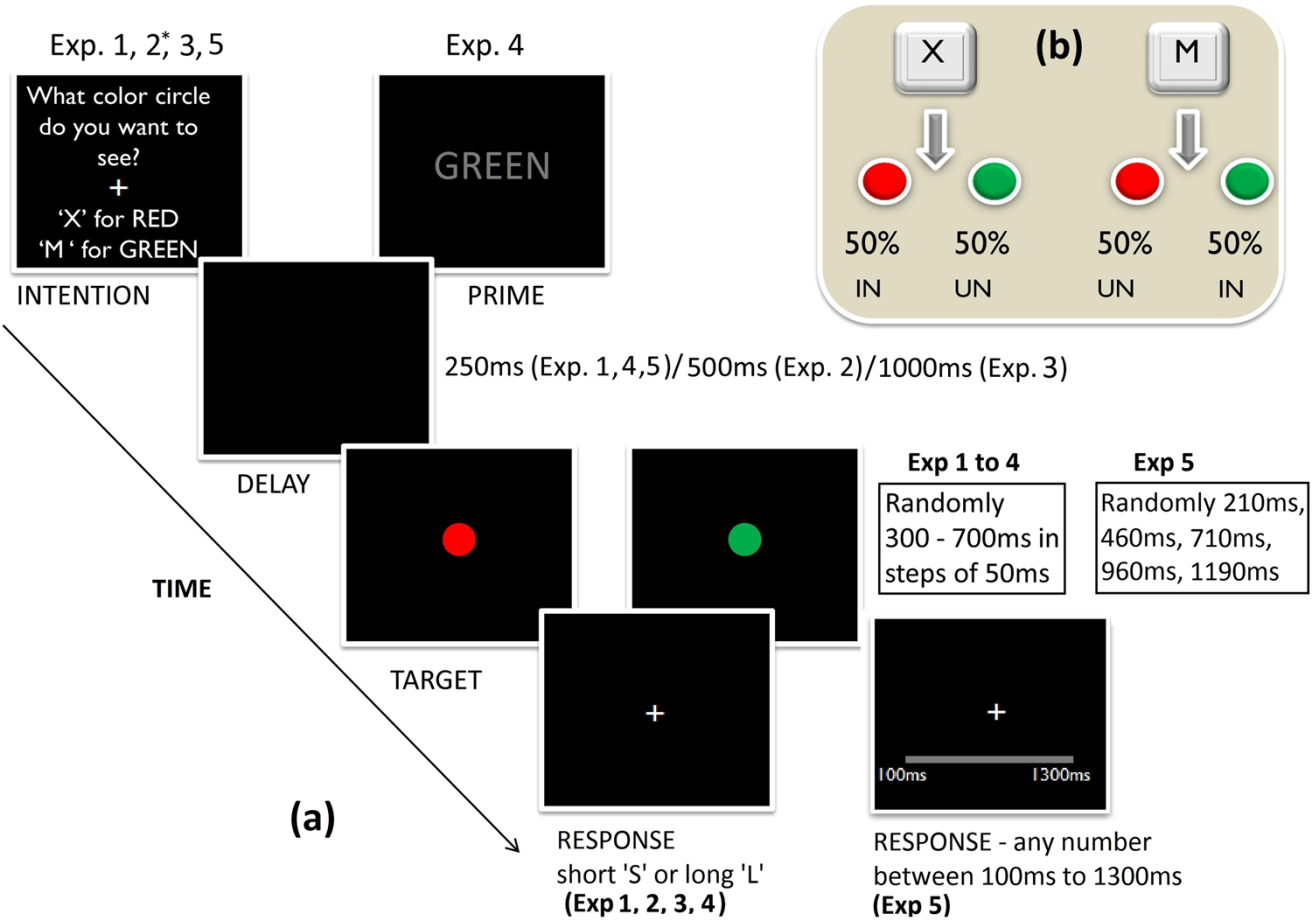
Experimental design. (**a**) Trial structure used in all the experiments. ‘*’ Indicates the experiment involved yellow and blue color circles instead of red and green. Before each trial participants selected what color circle they wanted to see by pressing the pre-assigned keys. The outcome appeared after a fixed delay in each experiment, but varied across the experiments [*250* *ms* in Exp 1, Exp 4, Exp 5; *500* *ms* in Exp 2; *1000* *ms* in Exp 3]. Randomly on half of the trials participants received the intended outcome and on the other half the unintended outcome. In Exp4, the participants had no choice but were forced to press the key based on the presented word, in this case on half of the trials the word matched the color of the circle (congruent), and on other half it did not match (incongruent). In Exp1, 2, 3 and 4 temporal bisection task was used, the target circle appeared for a variable duration (from 300 ms to 700 ms in steps of 50 ms), and participants reported the duration as either close to short (press ‘s’) or long (press ‘l’) anchor duration. In Exp5, a verbal estimation task was used, the target circle appeared for a variable duration (210 ms, 460 ms, 710 ms, 960 ms, 1190 ms), and participants used the computer mouse to report the estimated duration in the range of 100 ms to 1300 ms. (**b**) Distribution of intended and unintended outcomes.

To establish a link between IB and the perceived duration of the desired outcome, we studied the duration perception of the outcome for those conditions in which the effect of IB is well established. For example, typically the IB effect decreases as the delay between the action and its outcome increases^8, 16^, and the IB effect is present only for intention-based actions and not for stimulus-based actions^17^. To test whether an increase in action-outcome delay influences perceived duration of the outcome, we designed three experiments using a temporal bisection task in which participants received the action outcome after different delays of key press (250 ms-Exp 1; 500 ms-Exp 2 and 1000 ms-Exp 3). In all three experiments, participants first indicated which color circle they wanted to see; randomly in half of the trials participants received the intended outcome and in other half they received the unintended outcome (see Fig. 2). In the fourth experiment, we kept the action-outcome delay as 250 ms but made the task non-intentional i.e. instead of participants making an intentional selection, they were instructed to press the key based on the word (RED/GREEN) presented to them in each trial. In other words, participants made an instruction-based action and not an intention-based action. In case of a strong link between IB and the perceived duration of the action outcome, one should obtain temporal expansion for the intended outcome for the short action outcome delays (250 ms & perhaps 500 ms) and not for the long delay (1000 ms). And also, the temporal expansion should be not present for instruction-based action (Exp 4). While studies of *chronostasis* have demonstrated temporal expansion for the outcome of voluntary actions^18, 19^, they did not explicitly ask participants to intend or chose the outcome they wanted or manipulate the intentional nature of the outcome.

On the other hand in the time perception literature, amongst several models proposed to explain human timing behaviour, the “internal clock model”^20, 21^ is one of the most influential models. The clock model suggests a hypothetical internal clock consisting of a *pacemaker*- the pulse generator, which ticks at a particular rate. The *accumulator*, which accumulates the number of pulses or ticks corresponding to the stimulus duration. The *switch* or the *gate*, which connects the pacemaker and the accumulator. When the switch is closed, it allows the pulses to accumulate in the accumulator. Lastly, the *comparator* - which compares the accumulated pulses, with the number of pulses associated with standard duration stored in memory, to make the decision about the duration of the stimulus. Although the effects of many factors such as attention^22–25^, emotion^26, 27^, paradigm used^28, 29^ *etc*. influence time perception (see reviews^30–32^), the putative role of intentions on time perception is still lacking. It is believed that different factors influence time perception by changing different components of the internal clock. For example, factors like emotions^33–35^ or auditory clicks^36–38^ are believed to influence time perception by increasing the pacemaker speed. On the other hand factors like attention^39, 40^ are believed to influence time perception by affecting the opening and closing of the switch or gate (although there are debates regarding the role of attention, as some studies suggest attention also influences the pacemaker)^22^.

To identify whether any factor influences the pacemaker or the switch component of the internal clock, one can test the influence of that factor at multiple levels of test durations using tasks like verbal estimation. If the factor increases the pacemaker speed then the effect should increase in a multiplicative manner as a function of the increase in magnitude of actual duration, leading to differences in slope. If the factor influences the switch or gate then the effect (temporal expansion/compression) should remain more or less constant across different duration ranges, leading to differences in intercept. We performed experiment 5 to investigate whether intention influences duration perception by affecting the pacemaker speed or the switch/gating mechanism. We used a verbal estimation task, which makes it possible to measure the effect of intention for multiple durations and check for the presence of differences in slope or intercept. In addition, this experiment also allowed us to test whether the intentional influence on duration perception is present with a different time estimation task.

## Methods

### Ethics statement

All the experiments followed the guidelines approved by the Institutional Ethics Committee of University of Allahabad. All participants provided informed consent and were compensated with 50 INR.

### Participants

We recruited 81 healthy adult participants from Allahabad University with normal or corrected-to-normal vision (Exp 1 to 4, 14 participants each, and Exp 5, 25 participants). The following are the age and gender distributions for all five experiments; Exp 1 (22.9 years, 8 females), Exp 2 (21.1 years, 4 females), Exp 3 (20.7 years, 3 females), Exp 4 (19.7 years, 9 females) and Exp 5 (22.8 years, 15 females). For Exp 1–4 (temporal bisection task), we used Gpower software^41^ to calculate the apriori sample size for paired t-tests, at *α* = 0.05 and power = 0.8, the estimated effect size (*d*) = 0.9 was determined from a previous study^42^. For Exp 5 (verbal estimation task), participant number was decided by referring to a prior study that used a verbal estimation task with visual stimuli (effect size f = 2.7)^43^.

### Stimuli and Apparatus

All experiments were designed using E-prime software^44^ and run on a CRT monitor at a refresh rate of 85 Hz (Exp 5, 100 Hz). Participants sat 60 cm away from the monitor screen in a dark room. Stimuli consisted of colored circles. During the training phase of the temporal bisection task, a purple colored circle (diameter, 2.85^o^) was used. For the main experimental phase, red and green colored circles (diameter, 2.85^o^) were used (except Experiment 2, where yellow and blue circles were used).

## Procedure

### Temporal bisection task [Experiment 1–4]

We used a temporal bisection task^37, 45^ in experiments 1-4. The experiments consisted of two phases; first, the training phase and second, the experimental phase. In the training phase, each participant was trained to identify the short (300 ms) and long (700 ms) standard/anchor durations with above 80% accuracy. In the experimental phase, in each trial participants were asked to indicate whether they wished to see a red circle or green circle (except experiment 2, where yellow and blue color was used), by pressing a pre-assigned key specific to that color (except experiment 4, where participants were shown the word GREEN or RED on the screen and they were supposed to press the key corresponding to the word). To ensure they thought about their intentions and did not merely press a single key all the time, a bar indicating roughly how often they had chosen a particular color so far was shown on the side during the choice/intention slide. Participants were told that overall they could try to choose colors roughly equally and they could look at the bar, if they wanted to monitor it. Bars were present until participants made a choice. The bar was used in all the experiments in the study (except experiment 4 in which participants did not make an intentional choice). Please note no assumptions are made about free will or free choice; the bar is meant to be a loose constraint and as a cue to ensure they thought about the color they wanted to see in a particular trial.

Trial structure for all experiments is shown in Fig. 2. The target appeared 250 ms after the key press in experiment 1, and 4, whereas this delay was increased to 500 ms in experiment 2 and 1000 ms in experiment 3. The probability of getting the intended outcome was at chance (50%) so that the participant could not accurately predict whether the target would be the intended color or not. This was done to ensure that the effects are due to intention and not due to prediction. Out of 360 trials, randomly in 180 trials the participant received the intended or the desired outcome and in the remaining 180 trials (20 trials per level × 9 duration levels) they received the unintended or undesired outcome. Participants reported the duration of the circle (nine levels: 300–700 ms in steps of 50 ms) as closer to the short or long standard duration by pressing the corresponding key. Participants’ intention response i.e. what color they wanted to see, and their duration judgment response were recorded.

### Verbal estimation task [Experiment 5]

We used a modified verbal estimation task^36, 46^. Generally in this task participants are supposed to report the duration verbally or by pressing the number corresponding to the estimated duration. However, in this experiment we used a continuous analog scale. At the beginning of each trial, participants indicated their intention to see either a red circle or a green circle by pressing the predefined key. 250 ms after their key press, a target circle was presented at the centre of the screen whose duration was varied randomly amongst five levels [210 ms, 460 ms, 710 ms, 960 ms, 1190 ms]. Participants were supposed to give their estimate of the duration of the circle in milliseconds (between 100 ms to 1300 ms) using the mouse pointer. The resolution of the scale was 1 ms. The probability of getting the intended color circle was kept at chance level so that participants could not accurately predict the color of the circle. Overall there were 200 trials (40 trials × 5 blocks). Each block of 40 trials consisted of 4 repeats of each of the 5 duration levels for each condition (intended, unintended).

## Data Analysis

### Temporal bisection task

Individual participant’s data were first sorted into two conditions, (1) when the participant received the intended outcome (i.e. *Intended condition*) and (2) when they did not get the intended outcome (i.e. *Unintended condition*). Then the data for these two conditions were fitted with a psychometric function (Weibull function) using maximum-likelihood method in the psignifit version 2.5.6^47^ toolbox for MATLAB. In a typical psychometric plot the proportion of long response (y-axis) is plotted against the levels of actual duration (x-axis). The point of subjective equality (PSE - the duration that was perceived as closer to the short or long anchor duration 50% of the time) and difference limen (DL–the half of difference between p(long) at 75% and 25%) was computed. The PSE in the temporal bisection task is also called the bisection point (BP) and hereafter we use BP instead of PSE. A leftward shift of the psychometric functions reduces the BP value indicating longer subjective duration. DL represents the absolute sensitivity i.e. measure of precision; a smaller DL value indicates better precision. As suggested by Wichmann and Hill, deviance^47^, (D) is a better measure of goodness of fit, we used a deviance value above 95% confidence interval i.e. *Demp* > *D* ^*(0.975)^ to remove data with bad fit. Although most of the participants performed the task well, still there were five outliers (see supplementary material;1 participant in experiment 1, 2 participants in experiment 3 and 2 participants in experiment 4). In these experiments, since there were two within subject conditions, paired - t-test (two-tailed) was used to compare means. Opensource Jasp software^48^ was used to analyse data. We used Gpower^41^ to calculate effect size (dz).

### Verbal estimation task

Individual data were sorted by *intended* and *unintended* conditions. As no separate practice was given, block 1 data was not analysed, resulting in 160 trials [2 (Intended; Unintended) × 5 (levels of duration) × 16 repeats] for analysis. Mean estimates for each duration level were computed for each participant. To test whether intention influenced time perception in a multiplicative manner, we calculated the slope for both conditions using a linear regression model. Paired t-test analysis was done to compare the difference in slopes. A two way repeated measure ANOVA for 2 (Outcomes; Intended, Unintended) × 5 (Durations; 210 ms, 460 ms, 710 ms, 960 ms, 1190 ms) was performed using opensource Jasp software^48^. We used Gpower^41^ to calculate effect size (dz).

## Results

### Experiment 1

**Figure 3:**
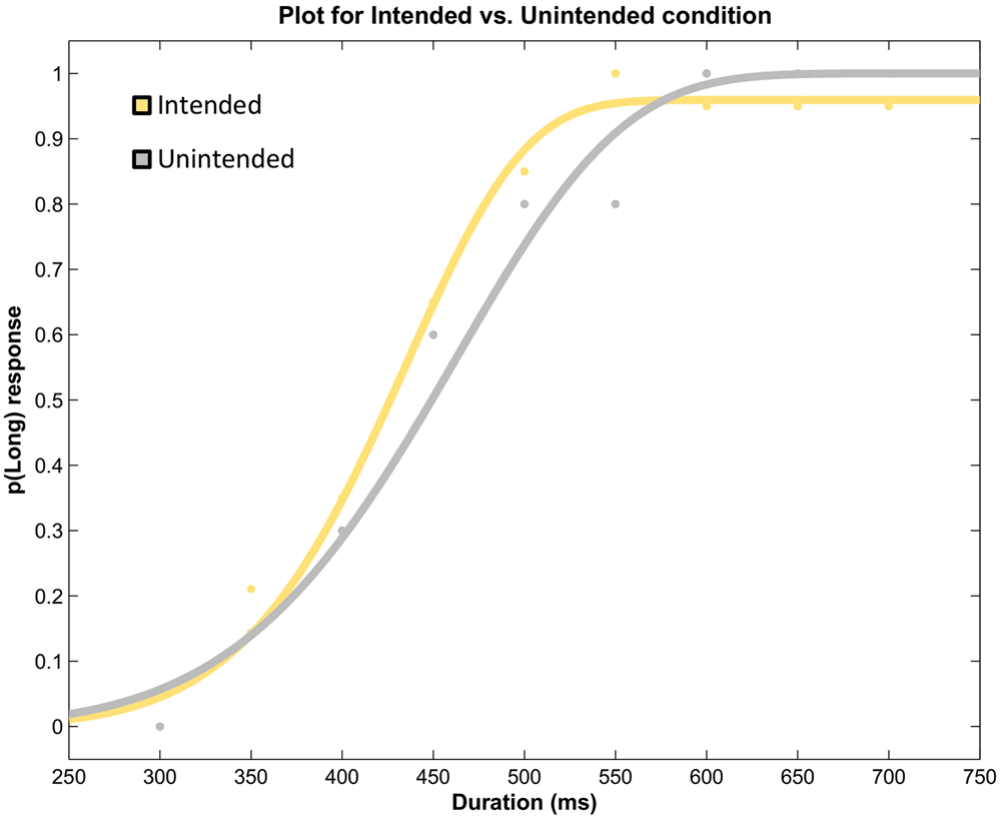
Plot depicting the fitted psychometric function (Weibull) for the intended and unintended conditions of a representative participant from Exp 1.

**Figure 4:**
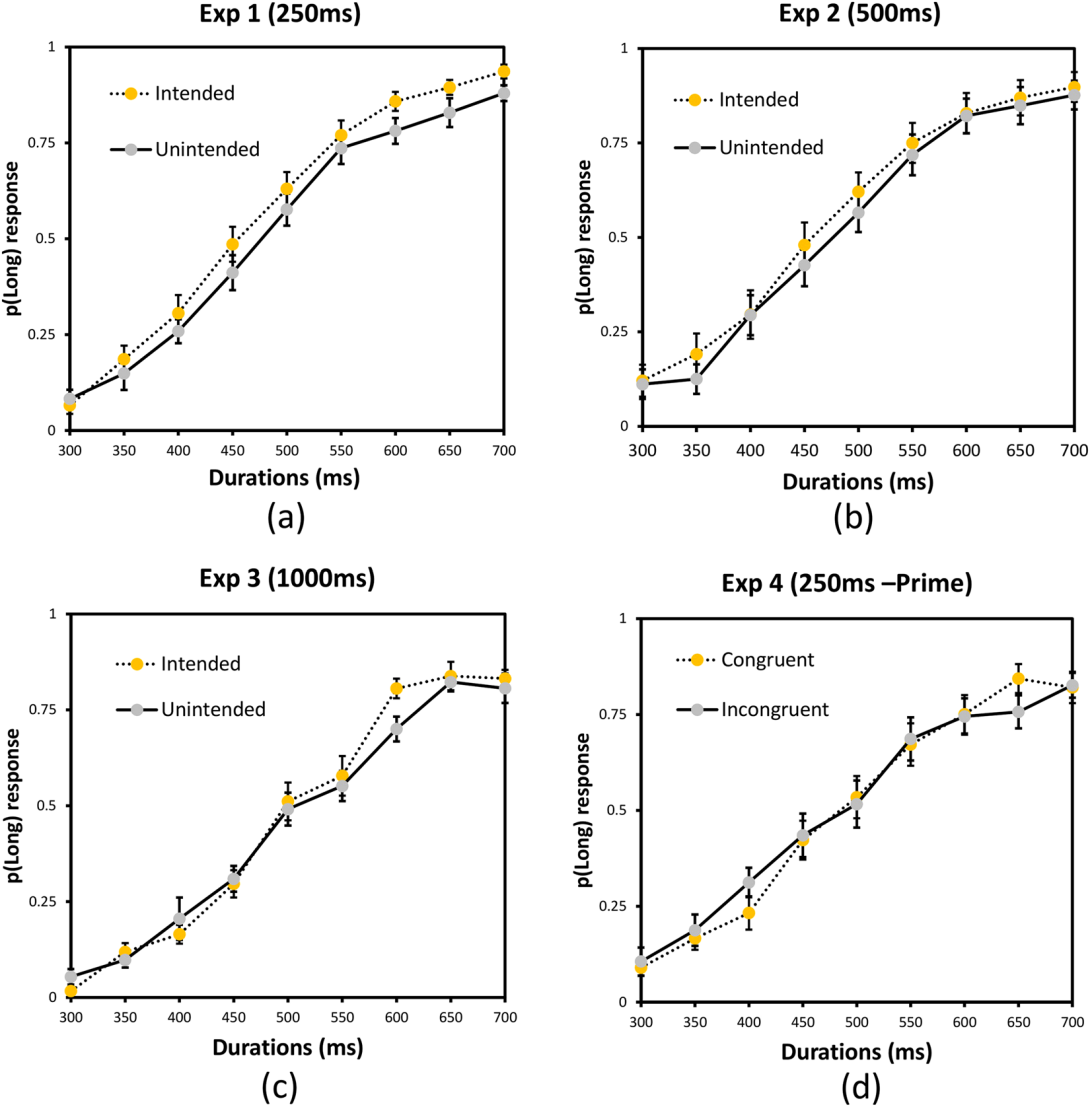
Plots depicting the proportion of long responses and standard error for each stimulus duration for intended and unintended conditions in (**a**) Exp 1, (**b**), Exp 2, and (**c**) Exp 3 as well as congruent and incongruent conditions in (**d**) Exp 4.

**Figure 5:**
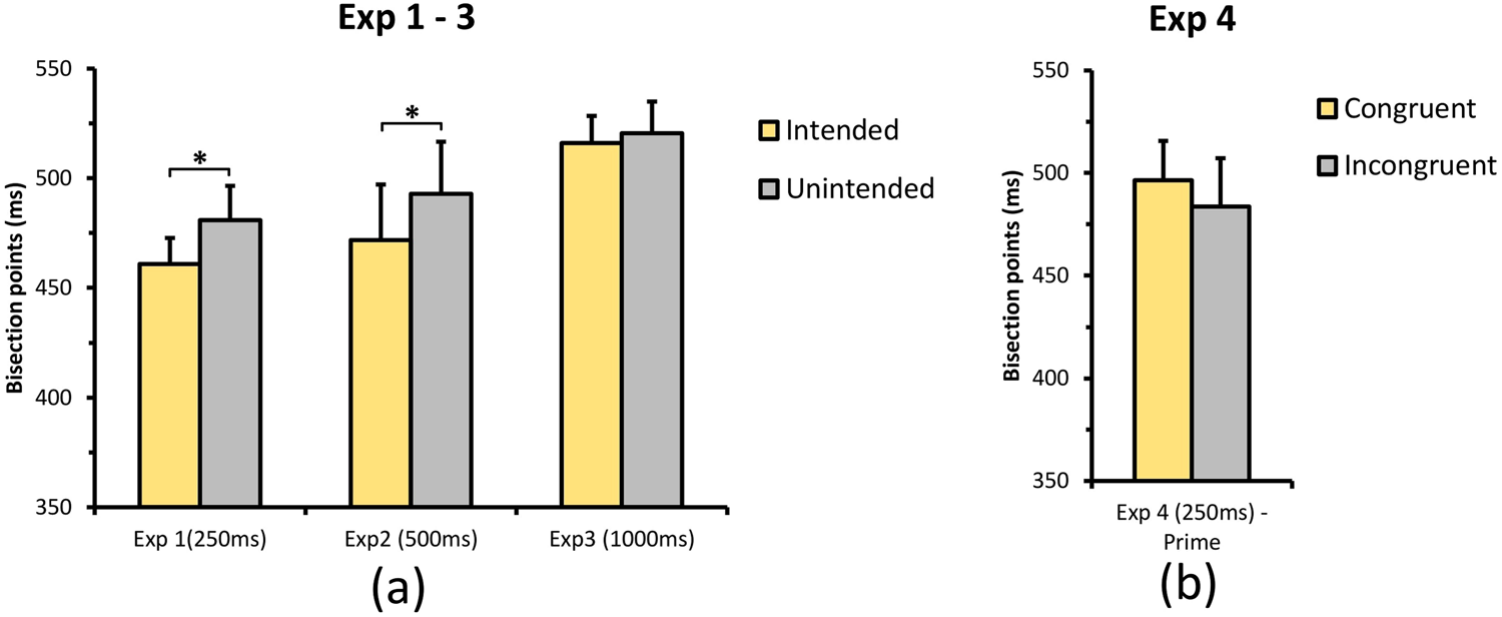
Results of four psychophysical experiments. (**a**) Represents the bar graph of BP (bisection point) for Experiments 1, 2 & 3, and (**b**) represents the bar graph of BP for two conditions in Experiment 4, where the prime (word) congruent or incongruent event appeared 250 ms after the key press. Error bar represents Standard Error (S.E.) and * indicates p < 0.05.

In this experiment, we tested whether perceived duration of the outcome is influenced by whether it was intended or not for the action-outcome delay of 250 ms. This delay has been used extensively in IB studies and it gives a strong IB effect. We predicted that if IB and duration are linked and onset of the intended outcomes is shifted more towards the action, at this delay, then the intended outcomes should be perceived longer compared to unintended outcomes. Figure 3 shows the fit for an individual participant. Analysis of BP values (see Table 1) showed that participants perceived the duration of the intended outcome to be significantly longer than the unintended outcome, *t*(12) = 2.346, *p* = 0.037, *Cohen’s d* = 0.651, mean difference = 19.94ms, 95% CI [1.424, 38.461]. Similarly, precision was better in the intended condition compared to the unintended condition, *t*(12) = 3.468, *p* = .005, *Cohen’s d* = 0.962, mean difference = 11.65 ms, 95% CI [4.332, 18.980]. The results show that participants perceived the intended event as longer compared to the unintended event (see Fig. 4(a) for p(long) responses and Fig. 5(a) for a bar graph of BP). A possible explanation is that intending a particular object or event leads to prior activation of the representation^49, 50^ of the event leading to expansion of time for the intended object.

**Table 1:** The Mean and Standard error (SE) of the bisection point (BP) and Difference limen (DL) for all four experiments as a function of intention (Experiments 1–3) or stimulus congruency (Experiment 4).

Measure	Condition	Exp 1		Exp 2		Exp 3		Exp 4	
		*Intended*	*Unintended*	*Intended*	*Unintended*	*Intended*	*Unintended*	*Congruent*	*Incongruent*
**BP (ms)**	*Mean*	460.81	480.75	471.70	492.81	516.02	520.49	496.38	483.62
	*SE*	11.81	15.66	25.38	23.843	12.332	14.408	19.18	23.50
**DL (ms)**	*Mean*	76.12	87.77	90.26	92.59	80.71	96.83	94.45	122.56
	*SE*	6.86	7.95	16.30	16.78	6.52	11.25	9.79	28.68

### Experiment 2

The results from experiment 1 showed that intention influences duration perception of colored circles. In experiment 2, we tested whether this effect would be present at an intermediate action-effect interval of 500 ms. We hypothesized that similar to IB, the effect of intention obtained in Experiment 1 would still be present at 500 ms but may be weaker. We also wanted to check whether an intention-induced temporal expansion effect (TEE) is present for different colored stimuli. Hence, yellow and blue color circles were used instead of red and green. Participants perceived the duration of the intended event to be significantly longer than the unintended event (see Table 1), *t*(13) = 2.948, *p* = 0.011, *Cohen’s d* = 0.788, mean difference = 21.11 ms, 95% CI[5.642, 36.580] (see Figs 4(b) and 5(a)). There was no difference in temporal precision between the two conditions, *t*(13) = 0.337, *p* = 0.742, *Cohen’s d* = 0.090, mean difference = 2.32 ms, 95% CI[−12.581, 17.229]. The results show that the effect of intention on duration is present at least till 500 ms and replicates our temporal expansion finding from Experiment 1 with different colors and response keys. However, the effect of intention on precision was not present at 500 ms indicating that the processes involved in changing temporal resolution may be effective only for very short action-effect intervals and a possible dissociation between the mechanisms that influence duration perception and temporal resolution. Although we predicted a weak effect of intentions on perceived duration at 500 ms delay compared to 250 ms delay, the magnitude of the effect was similar. While in general, the IB effect reduces with increase in action-outcome intervals, recent studies investigating the effect of action-outcome delay on the IB effect suggest that the effect of delay on IB depends on the time range and paradigm used^51, 52^. For example, Ruess *et al*.^51^ showed that IB increased with delay for the short time range but decreased for the long time range. In addition, another study using the method of constant stimuli to measure IB showed stronger temporal binding between active and passive key presses, for 600 ms compared to 250 ms^53^. In our study, we used a method of constant stimuli to measure perceived duration of the intended or unintended outcome and the specific parameters and method used may have resulted in a similar magnitude of intention induced temporal expansion in this experiment.

### Experiment 3

If intention leads to pre-activation of the representation of the intended outcome, then it is pertinent to ask about the temporal persistence of this representation and its effects on duration perception. With some exceptions^54^, the intentional binding effect is known to decrease as the delay between the voluntary action and its consequence increased to 1000 ms^8^. A similar fading effect with increase in delays between the action and its consequence also exists in sensory attenuation^55^, indicating that the representation of the intended outcome fades away with time. Hence, in Exp3 we increased the action-effect delay to 1000 ms to investigate the effect of intention on perceived duration at longer action-effect interval. If intentional binding and intention induced TEE have similar underlying mechanisms then there would be no effect of intention in this experiment, similar to Haggard *et al*.^8^. The effect of intention was not significant with BP (see Table 1), *t*(11) = 0.711, *p* = .492, *Cohen’s d* = 0.205, mean difference = 4.469 ms, 95% CI [−9.374, 18.312] (see Figs 4(c) and 5(a)) and with precision, *t*(11) = 1.439, *p* = 0.178, *Cohen’s d* = 0.415, mean difference = 16.116ms, 95% CI [−8.528, 40.761]. One possible explanation is that the activation of representation due to the formation of the intention has decayed by 1000 ms. The lack of effect is consistent with the lack of IB at similar action-effect intervals^8^. It should also be noted that different mechanisms are involved for sub-second and supra-second time ranges^56–58^ and the mechanisms underlying intentional subjective expansion of time are generally influenced by sub-second mechanisms and not supra-second mechanism even though a few studies have shown a temporal binding effect even at supra seconds delays^54^. Further studies are needed to understand the specific time course and the mechanisms involved in intention induced changes in temporal experience.

### Experiment 4

A possible explanation for intention-induced TEE is the prior activation of the representations associated with intended outcomes resulting in faster processing of intended compared to unintended outcomes. While this is plausible, prior activations can occur for multiple reasons and in this experiment we addressed whether mere activation caused by a prime leading to stimulus-based action would still be sufficient to produce effects similar to those obtained in Experiment 1. Intentional or voluntary actions are distinct from stimulus-based or instruction-based actions^59–61^. Intentional binding occurs for intention-based actions but not for stimulus-based actions^17^. We expected that as with IB, only intention-based action would produce temporal expansion of the outcome and activation based on intention is necessary to produce such an effect. We used color words to passively activate the associated color representation^62^.

As the normality assumptions was violated Wilcoxon signed rank test was used. The effect of prime congruency on BP (see Table 1) was not significant, *t*(11) = 56.00, *p* = 0.204, *Cohen’s d* = 0.476, mean difference = 11.5475 ms, 95% CI [−31.200, 7.155] (see Figs 4(d) and 5(b)). Precision was also not significantly different between prime-congruent and prime-incongruent conditions, *t*(11) = 34.00, *p* = 0.733, *Cohen’s d* = 0.269, mean difference = 5.55 ms, 95% CI [−22.122, 50.530]. The results indicate that potential activation of the representations by a prime is not sufficient to change the perceived duration and intentional activation might be needed to alter perceived duration of the intended outcome.

### Experiment 5

**Figure 6:**
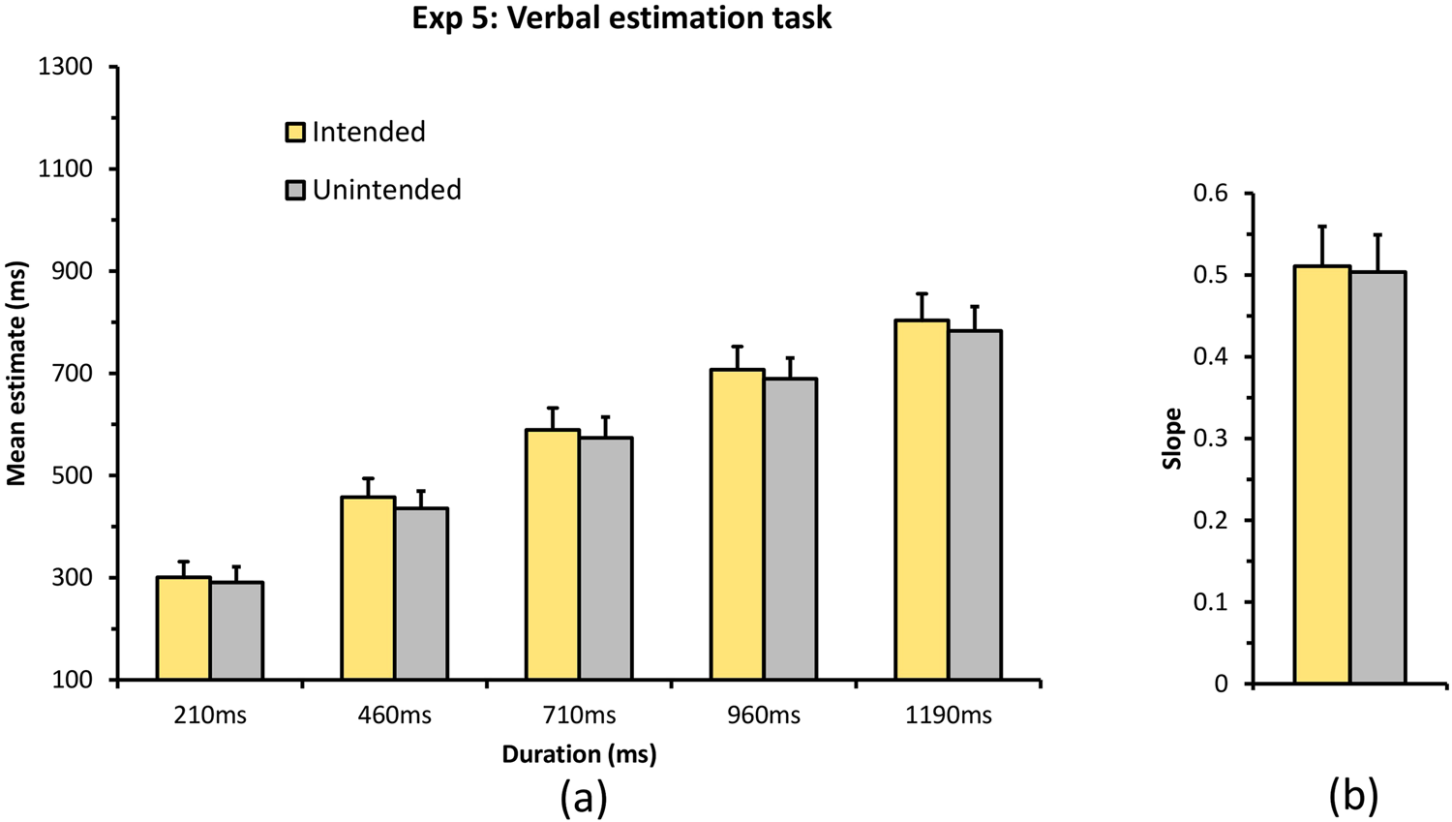
Results for Experiment 5 using a Verbal estimation task. (**a**) Plot represents the mean estimated duration for intended and unintended conditions against the actual presented duration. (**b**) Plot represents the mean slope for intended and unintended conditions. Error bar represents Standard Error (S.E.).

A repeated measures ANOVA treating outcome (*Intended, Unintended*) and duration (*210* *ms, 460* *ms, 710* *ms, 960* *ms, 1190* *ms*) as within subjects factors was performed on the mean estimates (see Fig. 6). Results showed a significant main effect of Intention [*F*(1, 24) = 8.45, *p* = 0.008, *η* ^*2*^ = 0.261] indicating that participants perceived the intended outcome to be longer compared to unintended outcome. Greenhouse-Geisser correction was applied wherever sphericity was violated. Main effect of Duration [*F*(1.29, 31.17) = 102.74, *p* <0.001, *η2* = 0.811] was also significant indicating that participants were able to discriminate between different duration levels. The interaction between intention and duration was not significant. Slopes were obtained for intended and unintended conditions by fitting a linear regression line between estimates and the actual duration. Paired t-test for individual slopes for intended and unintended condition was not significant [*t*(24) = 0.687, *p* = 0.499, *Cohen’s d* = 0.137] indicating that there was no *“slope effect”*. This experiment suggests that intention does enhance the duration of the outcome but the magnitude of this enhancement does not increase drastically with duration magnitude, indicating that the mechanisms via which intention affects duration perception cannot be fully explained by an increase in pacemaker speed. Further studies are needed to understand the detailed mechanisms involved in intention-induced temporal expansion.

## Discussion

We investigated the role of intention on time perception. Results showed that an intended event is perceived longer when it appears after a shorter delay (250 ms-Exp 1 or 500 ms-Exp 2) but not when it appears after longer delay (1000 ms-Exp 3). Furthermore, Exp 4 confirmed that this effect was due to ‘intentions’ since the effect was not present for explicit instruction-based action. Results from Exp 5, suggest that intentions might not increase the pacemaker speed of the internal clock, but rather influence the *switching*/*gating* mechanism. To our knowledge this is the first study to investigate the effect of intentions (what one wants to see) on duration perception of perceptual outcomes.

The presence of intention-induced temporal expansion at shorter action-effect intervals and only for intentional actions (in contrast to instruction-based actions) mirrors the findings on IB^8, 17^ indicating common mechanisms. With respect to the question of whether in the case of IB the entire outcome event or only the onset shifts towards the action, our results support the *only onset shifts* hypothesis. A study by Kanai and Watanabe^63^ also supports the onset shifting hypothesis which suggests that the visual onset shift leads to expansion of subjective time. Moreover, by assuming such a mechanism, both IB and temporal expansion of the outcome (also chronostasis) can be explained in a single common framework (see Fig. 1). It has been argued that causal relationships between an action and its outcome results in IB^12, 64^. We do concur that causal relationships play an important role in IB and more generally in agency. It is possible and likely that casual relationships between intention and outcome also would produce a similar or larger temporal expansion effect. However, in our study, the outcomes are not predictable. Hence, causal relationships per se cannot explain the observed effects of intentions on the perceived duration of the outcome. It should be noted that one can desire an event independent of the probability of the outcome of that event. One can desire low-probability events (for example, lotteries) and desire a particular event even when there is uncertainty (probability information is not known). One could also argue that participants could have an illusion of causality, but it is likely that with such a large number of trials one would learn that the outcome is not predictable. More importantly, the probability relationship between the intention and the outcome is provided explicitly to the participants. We consider causality or falsely perceived causality as unlikely to explain the results on subjective duration of the outcome in the current study. In addition, the effects were not present when participants perform the action based on instruction (as in Exp 4) indicating that forming an intention is important for the effect obtained in experiments 1, 2 and 5.

The above results can be explained by a variation of pre-activation accounts^8, 42, 50^ wherein intending an event activates its representation. Such intention-induced pre-activation would enable faster processing of the intended event leading it to reach awareness earlier compared to the unintended event. As the awareness of the intended event is marked earlier than the unintended event, this shift in onset would lead to expansion of perceived duration of intended compared to unintended events (see Fig. 1). Recently, Press *et al*.^42^ showed that participants perceived the duration of an avatar-hand (on screen) to be longer when its finger movement matched with the participant’s finger movement possibly due to pre-activation of action-related consequence. They instructed participants about which finger to lift; participants did not choose what they wanted to see. Generally, pre-activation requires a strong (usually predicted or causal) action-effect relationship, so that when an action is prepared/executed, it also activates the associated outcome representation^42, 49, 50, 65^. Results from both behavioural and electrophysiological studies suggest that activations of self-induced expectations are stronger than cue-induced expectations^60, 66, 67^. We suggest that even without a strong action-effect contingency (predictive relationship), strong pre-activation occurs when an agent ‘intends’ an event. This could be somewhat similar to the cases where representations are activated during mental imagery even without actual stimuli and many common brain regions are activated during imagery, which are active during perception^68–72^. For example, thinking about a face activates the fusiform face area whereas thinking about a house activates the parahippocampal place area^68^.

Other possible mechanisms that could explain our results include (i) event shifting or recalibration (ii) attention capture by voluntary actions, and (iii) and increased arousal by voluntary actions. Recalibration accounts propose that the brain recalibrates the time between the action and its outcome^15^ and IB has been suggested to occur due to the shifting of the entire event towards the action rather than due to temporal compression. If the *whole* outcome event is shifted due to intention, then recalibration would not be able to explain the temporal expansion effect found in our study. However, if recalibration happens only for the *onset* of the intended outcome event, then it could still be used to explain our effect.

Attention is known to expand temporal perception^22, 23^, but a recent study^73^ suggested that the outcome does not capture attention when there is no predictive relationship between the action and its outcome. Given that the intended outcome was not predictable in our experiments, it is less likely that attention capture per se caused the intention-induced TEE.

Voluntary action can increase arousal and accelerate the ‘internal clock’, leading to subjective temporal expansion of the consequences of voluntary actions^19^. In our study, participants performed voluntary action in both the intended and unintended conditions. If voluntary action accelerates the internal clock, then it would be present for both the conditions and would not be able to explain the intention-induced TEE. The intention or desire to take an action, and the matching of that intention with the expected outcome is important for intention-induced TEE.

It is plausible that the occurrence of an intended event led to more attention being paid to the event, or an increase in arousal given that the intended or desired event has occurred. This could potentially result in increasing the speed of the internal clock, thus leading to subjective expansion of time. A recent study by Failing & Theeuwes^74^, showed that the duration of high-reward stimuli is perceived longer compared to low-reward stimuli. As the reward system is linked to dopamine, and dopamine is known to accelerate pacemaker speed, such rewarding or salient stimuli are known to elongate temporal perception. Recently, Terhune *et al*.^75^ reported that an increase in striatal dopamine indicated by spontaneous blinks, may be responsible for the moment to moment variation in subjective time. While a potential mechanism like increase in dopamine is plausible when a participant receives the intended outcome, currently there is no such evidence for intention-induced increase in dopamine or arousal or attention, specific to the intended outcome. Further studies are needed to investigate whether attention and arousal mechanisms are influenced by intentions and mediate the effects of intentions on perception.

The findings of the study also have implications for theories of time perception. Most of the theories of time perception focus on content^40, 76–80^ but not on processes of intention and volition. The findings indicate that whether an event was intended influences perceived duration in addition to the content of the event itself. In conclusion, our time perception is not only based on what we see but also on whether we saw what we wanted to see.

## Electronic supplementary material

Supplementary information
