# Supplementary material for: Intended outcome expands in time
Source: Sci Rep. 2017 Jul 24;7:6305. doi: 10.1038/s41598-017-05803-1 (PMC5524699; doi:10.1038/s41598-017-05803-1)
Supplement: Supplementary file 1 — Supplementary information [file 41598_2017_5803_MOESM1_ESM.doc]

Intended outcome expands in time

Mukesh B. Makwana & Narayanan Srinivasan

Centre of Behavioural and Cognitive Sciences, University of Allahabad, Allahabad, India

**Supplementary material**

Deviance (D) measure was used to assess goodness-of-fit of the psychometric function (weibull), fitted using maximum likelihood method in psignifit 2.5.6 matlab toolbox (see Wichmann & Hill, 2001a). Based on the criteria suggested by Wichmann and Hill, i.e. Deviance cumulative probability estimate (cpe) > 0.975 indicates bad fit; overall we found five bad fits. The following are Dcpe values for Exp 1-4. The highlighted value indicates Dcpe > 0.975, which were removed from further analysis.

|  | Exp1 | | Exp2 | | Exp3 | | Exp4 | |
| --- | --- | --- | --- | --- | --- | --- | --- | --- |
| Subjects | Intended | Unintended | Intended | Unintended | Intended | Unintended | Congruent | Incongruent |
| 1 | 0.9955 | 0.7505 | 0.9475 | 0.952 | 0.9695 | 0.041 | 0.213 | 0.097 |
| 2 | 0.867 | 0.7425 | 0.924 | 0.0805 | 0.1035 | 0.663 | 0.569 | 0.6615 |
| 3 | 0.295 | 0.728 | 0.0035 | 0.7795 | 0.7955 | 0.745 | 0.998 | 0.862 |
| 4 | 0.95 | 0.886 | 0.6235 | 0.86 | 0.858 | 0.8585 | 0.6605 | 0.3765 |
| 5 | 0.4345 | 0.8115 | 0.4355 | 0.5985 | 0.298 | 0.823 | 0.05 | 0.6695 |
| 6 | 0.9695 | 0.184 | 0.76 | 0.6485 | 0.9545 | 0.364 | 0.1375 | 0.7745 |
| 7 | 0.3245 | 0.9125 | 0.338 | 0.893 | 0.9815 | 0.009 | 0.043 | 0.9805 |
| 8 | 0.85 | 0.0555 | 0.7475 | 0.6655 | 0.884 | 0.9865 | 0.9695 | 0.365 |
| 9 | 0.926 | 0.854 | 0.642 | 0.7575 | 0.6845 | 0.828 | 0.332 | 0.3145 |
| 10 | 0.3345 | 0.2195 | 0.054 | 0.0665 | 0.474 | 0.2105 | 0.2605 | 0.733 |
| 11 | 0.0935 | 0.5665 | 0.4345 | 0.364 | 0.949 | 0.3365 | 0.9425 | 0.94 |
| 12 | 0.018 | 0.2075 | 0.563 | 0.7505 | 0.2855 | 0.6365 | 0.734 | 0.294 |
| 13 | 0.43 | 0.254 | 0.1255 | 0.9165 | 0.9435 | 0.3945 | 0.8735 | 0.047 |
| 14 | 0.187 | 0.4735 | 0.0485 | 0.32 | 0.0425 | 0.776 | 0.835 | 0.081 |
